# Artificial intelligence framework for modeling and predicting crop yield to enhance food security in Saudi Arabia

**DOI:** 10.7717/peerj-cs.1104

**Published:** 2022-09-30

**Authors:** Mosleh Hmoud Al-Adhaileh, Theyazn H.H. Aldhyani

**Affiliations:** 1 Al Bilad Bank Scholarly Chair for Food Security in Saudi Arabia, The Deanship of Scientific Research, The Vice Presidency for Graduate Studies and Scientific Research, King Faisal University, Al Ahsa, Saudi Arabia; 2 Deanship of E-learning and Distance Education, King Faisal University, Al-Ahsa, Saudi Arabia; 3 Applied college in Abqaiq, King Faisal University, Al-Ahsa, Saudi Arabia

**Keywords:** Crop yield, Artificial intelligence, Prediction model, Food security

## Abstract

Predicting crop yields is a critical issue in agricultural production optimization and intensification research. Accurate foresights of natural circumstances a year in advance can have a considerable impact on management decisions regarding crop selection, rotational location in crop rotations, agrotechnical methods employed, and long-term land use planning. One of the most important aspects of precision farming is sustainability. The novelty of this study is to evidence the effective of the temperature, pesticides, and rainfall environment parameters in the influence sustainable agriculture and economic efficiency at the farm level in Saudi Arabia. Furthermore, predicting the future values of main crop yield in Saudi Arabia. The use of artificial intelligence (AI) to estimate the impact of environment factors and agrotechnical parameters on agricultural crop yields and to anticipate yields is examined in this study. Using artificial neural networks (ANNs), a highly effective multilayer perceptron (MLP) model was built to accurately predict the crop yield, temperature, insecticides, and rainfall based on environmental data. The dataset is collected from different Saudi Arabia regions from 1994 to 2016, including the temperature, insecticides, rainfall, and crop yields for potatoes, rice, sorghum, and wheat. For this study, we relied on five different statistical evaluation metrics: the mean square error (MSE), the root-mean-square error (RMSE), normalized root mean square error (NRMSE), Pearson’s correlation coefficient (*R*%), and the determination coefficient (*R*^2^). Analyses of datasets for crop yields, temperature, and insecticides led to the development of the MLP models. The datasets are randomly divided into separate samples, 70% for training and 30% for testing. The best-performing MLP model is characterized by values of (*R* = 100%) and (*R*^2^ = 96.33) for predicting insecticides in the testing process. The temperature, insecticides, and rainfall were examined with different crop yields to confirm the effectiveness of these parameters for increasing product crop yields in Saudi Arabia; we found that these items had highest relationships. The average values are *R* = 98.20%, 96.50, and 99.14% with for the temperature, insecticides, and rainfall, respectively. Based on these findings, it appeared that each of the parameter categories that are considered (temperature, pesticides, and rainfall) had a similar contribution to the accuracy of anticipated yield projection.

## Introduction

Because of rising concerns about food security, crop output prediction is becoming more relevant. Early crop production forecasts can significantly contribute to the reduction of famine by predicting the availability of food for the rising global population. Increased food yields are a possible way to end global hunger, which is one of the most severe problems of our day. According to the World Health Organization, an insufficient food supply still exists for 820 million people throughout the world, despite recent improvements. The United Nations’ Sustainable Development Goals (SDGs) aim to eradicate hunger, achieve food security, and promote sustainable agriculture by 2030, with a particular emphasis on agriculture (WHO, 2021). By 2050, the Food and Agriculture Organization of the United Nations (FAO) predicts a 60% rise in food demand to feed the world’s population of 9.3 billion people (UN, 2021). Crop production forecasting, as a result, may provide critical information for building a feasible approach to fulfilling the goal of ending hunger (Kheir et al., 2021).

Improvements in agroclimatic conditions, rainfall persistence, soil quality, and other infrastructure are all critical aspects in ensuring that Norway’s agricultural output generation is sustainable (Eltun, Korsaeth & Nordheim, 2002). Due to the rapid growth in the world’s population, farmers are faced with a huge challenge in producing more amounts of higher-quality grains (Klaus, 2005). It is our goal in this research to examine agricultural production prediction at the farm scale. According to our expectations, it will give farmers useful insights on the specific kinds and amounts of crops that will be available throughout certain seasons depending on geographical locations and other environmental parameters. Additional benefits include increased food security and the facilitation of decision making at different administrative levels.

When it comes to crop production, several variables should be considered, making it challenging to develop a good forecast model using standard approaches. In recent years, however, advances in computer technology have made the creation and training of a new technique for agricultural production prediction a possibility. As a result of its diverse data technologies and high-performance processing capabilities, deep learning is an important approach that is widely employed in the agricultural area. One subfield of machine learning is known as “deep learning”, and it is characterized by the use of multiple layers of neural networks that are capable of gaining knowledge from inputs that are both unstructured and unlabeled. The learning may be supervised, semi-supervised, or unsupervised, depending on the learning environment. Sarker (2021) pointed out that deep learning approaches are focused on learning abstract characteristics from big datasets, as opposed to traditional machine learning techniques. To effectively anticipate crop production, it is necessary to have a thorough understanding of the relationships that exist between functional qualities and interacting variables. Such correlations require the use of large datasets and high-efficiency algorithms, both of which may be accomplished *via* the use of deep learning  (Tranfield, Denyer & Smart, 2003; Kitchenham & Charters, 2007).

Machine learning has been an extensively investigated area during the last decade, and it is now being used to forecast and increase agricultural yield outputs all around the globe (Klompenburg, Kassahun & Catal, 2020; Shao, Ren & Campbell, 2018). Numerous studies have shown that crop production prediction models developed at the county level are well suited for use at the regional or national level. Farm-scale production prediction, on the other hand, has only been the subject of a few studies (Wang et al., 2018). Because of a lack of support for sustainable agriculture and the high cost of obtaining satellite photos, farm-scale ground-truth data are scarce (Lobell et al., 2015; Basso, Cammarano & Carfagna, 2013). These barriers, however, seem to be fading in the agricultural sector. Since 2017, complete agricultural reports, including farm-scale information, have been made accessible to the public in Norway. Copernicus, the European Union’s Earth observation program, provides high-resolution satellite photographs, which may be accessed *via* the Copernicus website.

Considerable improvement is still needed in terms of a robust and an appropriate approach to creating an accurate and rapid learning framework, even though artificial intelligence (AI) technologies have resulted in important applications for crop development. A novel neural network (ANN) model is developed to satisfy the above-mentioned needs. Input–output dynamics are well-represented by ANNs. The main contributions of the presented study are listed below:

•  By using AI models, it is feasible to evaluate how temperature changes, rainfall amounts, and insecticides all affect yield. A series of yield data, which was influenced by agricultural measures and external meteorological conditions in Saud Arabia, was initially used in the study to establish its applicability.

•  The multilayer perceptron (MLP) model was used to predict future values for different crop yields, such as potatoes, rice, sorghum, and wheat.

•  Using a congruence correlative empirical orthogonal function, the AI approach reduces the time needed to predict crop yields. Data are analyzed using an AI model to identify useful or insignificant elements. Instead of using all of the dataset’s attributes for crop production prediction, only the most relevant features are employed.

•  Different measures are used to evaluate the suggested MLP method’s performance, and the findings reveal that it outperforms the other baseline techniques.

## Related Work

Traditional methodologies, such as the static regression approach and the mechanistic approach, have limited application and uncertainty (Horie, Yajima & Nakagawa, 1992) and make developing a reliable crop production forecast model challenging. Several studies have used machine learning to forecast agricultural production. Machine learning algorithms, unlike traditional statistical models, interpret the output variable, crop production, as an implicit function of the input parameters, such as weather and soil conditions, which might be complex (Jeong et al., 2016). Unfortunately, the nonlinear link between input and output variables is not captured by supervised learning algorithms in machine learning  (Islam et al., 2021b). However, technological improvements in recent years have made it feasible to construct an enhanced crop production forecast model based on deep learning, which is now under development. In machine learning, deep learning is a family of techniques that employs hierarchical structures to connect layers of data. Its ability to evaluate both unlabeled and unstructured data distinguishes it from other standard machine learning methods (Islam et al., 2021a). In the agricultural area, deep learning is widely utilized because it can analyze large datasets, understand the links between numerous factors, and employ nonlinear functions. Deep learning is particularly useful since it can analyze and learn the correlations between many variables. These unsupervised techniques may be used to extract features from large datasets in an unsupervised setting. While standard machine learning algorithms perform better in feature extraction, deep learning approaches outperform them (LeCun, Bengio & Hinton, 2015). Because an accurate crop yield forecast is dependent on the elements that influence crop development, deep learning has a great capacity to extract features from existing data, which is particularly useful in agriculture.

Deep neural networks (DNNs) are comprised of a set of nonlinear layers that, at each layer, transform the untested input data into an extracted form, thereby forming a network. To identify the nonlinear associations between input and response variables, DNNs with several hidden layers are required. But they are challenging to train and need the use of freshly discovered hardware and optimization methods (Goodfellow et al., 2016). As a result, increasing the number of hidden layers may be useful, but it comes with certain limitations that can be overcome by using certain strategies. A technique known as residual skip connections for the network (Khaki, Wang & Archontoulis, 2020a; Szegedy et al., 2015) has been shown to be effective in alleviating the vanishing gradient issue in deeper neural networks. Furthermore, the performance of deep learning systems has been enhanced by the use of numerous techniques, such as stochastic gradient descent (SGD), batch normalization, and dropouts.

In numerous exciting disciplines, such as powder metallurgy and material analysis (Cherian, Smith & Midha, 2000; Smith, German & Smith, 2002), the applications of artificial neural networks (ANNs) have been investigated. It is stated in Sanzogni & Kerr (2001) that a feedforward ANN with a postprocessing polynomial may be used to forecast milk production on dairy farms. M Korosec and colleagues provide a neuro-fuzzy model that relies on the idea of “product manufacturability” to define and accept the degree of “pretentiousness-machining” difficulty (Korosec, Balic & Kopac, 2005; Hu et al., 2009; Agrawal & Schorling, 1996). Because buyers and sellers are impacted by a variety of unforeseen variables that interact in an intricate manner, accurately forecasting the global rice trade is always difficult. The dependability of ANNs is compared to that of ARIMA models in a study by Co & Boosarawongse (2007), and an exponential smoothing is predicted for Thailand’s rice exports.

Several research studies have been conducted on Chinese fruit production, but only a few have been published in the literature  (Ali & Imran, 2020; Vakil-Baghmisheh & Pavešić, 2003). Friis & Nielsen (2016) suggested banana plantation investments in the United States (Luang Namtha Province, Laos). Viani et al. (2017) developed an autonomous wireless decision support system for water agriculture that was integrated into the network gateway. Calculating the moisture content (MC) and humidity of agricultural goods using a capacitive sensor has been specified in some regions (McIntosh & Casada, 2008). Ochiai et al. (2011) obtained data on agricultural applications using DTN-based sensor gathering.

Machine learning and deep learning methods are increasingly being used to remotely sense data to evaluate and forecast various agricultural yields (You et al., 2017; Cai et al., 2019). Several researchers have suggested that nonlinear approaches can surpass linear models for predicting and estimating yields from remotely sensed data (Johnson, 2014). Climate and management are two important aspects that might have an effect on crop phenology. Phenology is described as “the study of the timing of recurrent biological occurrences”. A crop’s phenology shifts from one season to the next as a result of changes in the climate and agricultural practices (Nejedlik, Oger & Sigvald, 2021). For Jiang et al. (2019), the objective of their project was to determine whether a phenology-based LSTM model could be utilized to estimate maize yields. During the course of the growing season, a crop of corn goes through a total of six unique stages of development, some of which include being planted, emerging, silking, doughing, denting, and maturing. Following this protocol, growing corn was divided down into five different stages in this study. Each stage of development corresponds to a single time step in the LSTM (from seedling to emergent, emerging to silking, silking to dough, dough to dented, and dented to mature). Throughout each and every time step, we were required to analyze three weather features in addition to a single vegetation index. The Wide Dynamic Range Vegetation Index (WDRVI) is a vegetation index that is comparable to the National Vegetation Data Index (NDVI). When a high density of biomass exists, the saturation effect is less of a problem (Walker, Olesen & Phillips, 2001). This outcome is better than the RuleQuest Cubist (0.96), although it is impossible to directly compare since the number of seasons included in the training and the seasons in which the training took place are not the same (Nejedlik, Oger & Sigvald, 2021).

Crop yield prediction is another area where DNNs have been extensively employed, either alone or as part of a multimodal combination. To determine the most accurate model for predicting winter wheat production, Cao et al. (2020) compared DNN to machine learning techniques, such as a support vector machine (SVM) and random forest. The DNN technique to estimate biomass was examined by Jin et al. (2019). It was initially successful with 15 vegetative indicators, but the accuracy of the DNN’s biomass estimate increased when the leaf area index (LAI) and 15 indices were used together. For end-of-season and within-season crop production predictions, a binarized neural network (BNN) performed better than machine learning techniques. Ma et al. (2021) showed that using a region-based convolutional neural network (R-CNN) reduced the number of possible areas for object identification while maintaining high accuracy. For strawberry flower and fruit detection, researchers examined R-CNN, fast R-CNN, and faster R-CNN. Faster R-CNN had the best performance and required the least amount of time to train. For multimodal data mining to be effective, it must be possible to consistently represent intermodality and cross-modality in the global space in which the data are integrated (Mkhabela et al., 2011; Barbosa et al., 2021; Bronstein et al., 2010; Poria et al., 2017). Multimodal data fusion is a key component of this process. Using 3D-CNN and Conv-LSTM networks together, Gavahi, Abbaszadeh & Moradkhani (2021) came up with a deep yielding technique. A DNN architecture with feature fusion at the input and intermediate levels was utilized to forecast agricultural production by Maimaitijiang et al. (2019). According to the suggested multimodal deep learning framework, the intermediate-level feature fusion DNN framework outperformed its input-level feature fusion DNN framework in terms of prediction accuracy, spatial adaptability, and resilience. Multimodal deep learning was employed by  Danilevicz et al. (2021) by integrating tab-DNN, sp-DNN, two linear layers of fusion, and ReLU. The weights from the last layers of tab-DNN and sp-DNN were combined for the fusion module input. The method worked effectively for predicting early crop yields. Machine learning, deep learning, and ensemble approaches stack many networks on top of one another and employ the characteristics gleaned from each one. Sun et al. (2020) combined many models into one network and stacked it using convolution, pooling, and fully connected networks.

In conclusion, we will conduct a literature study on the use of artificial intelligence techniques in crop yield prediction. Because it has the capacity to reveal the existing research gaps in a certain area of artificial intelligence methodology and aids us in assessing those gaps, the justification for the practice of doing a literature review is that it is important to do so. the influence that vegetation indicators and environmental conditions have on the growth of crops. This research is viewed from a different angle thanks to the literature review, which analyzes the benefits of the study. In the process of estimating crop yields using artificial intelligence models, the most appropriate technology is remote sensing. predicated on the necessities of data collecting, as well as the numerous factors that play a role in crop yield prediction In addition, information on temperature, precipitation, soil conditions, and pesticides are all taken into account when attempting to forecast crop yield. There is less research being done to uncover individual traits that have a substantial impact on crop yield prediction as a result of the fact that crop yield prediction currently makes use of a large number of features. As a result, in-depth study is required in order to gain a better overview of these variables and factors influencing crop production forecast than can be achieved through modeling based on previous research.

## Materials and Methods

**Figure 1:**
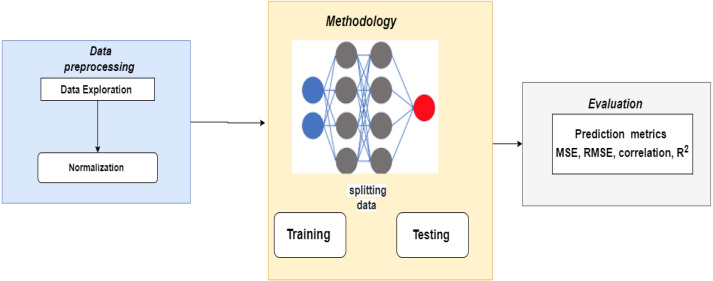
Framework of the proposed system.

Figure 1 displays the framework of the proposed system to predict crop yields in Saudi Arabia. In this research, the ANN model was proposed to predict future values.

The current research is being directed by the following research questions, which have been prepared to serve as a guide.

Q1. What artificial intelligence approaches are used for crop yield prediction?

Q3. How develop artificial intelligence for predicting future crop yield in Saudi Arabia?

Q4. What are important environmental parameters for increasing crop yield in Saudi Arabia

This study investigates the feasibility of developing neural network models that can make use of data on crop yields to make farm-scale yield predictions in Saudi Arabia with the goal of ensuring continued access to nutritious food supplies. Through the utilization of various AI technologies, the primary purpose of this work is to make projections about the future values of important crop yields in Saudi Arabia. In addition, determining the relationship between environmental factors such as precipitation, temperature, and the use of insecticides and the yields of various crops, such as potatoes, rice, sorghum, and wheat, is another important step.

### Datasets

Predicting crop productivity is a significant issue in agriculture. Producing high-quality food for human consumption heavily relies on a variety of factors, chief among them being the weather (such as temperature and rainfall), insecticides, and historical data on crop productivity. In the end, we all need the same fundamental essentials for survival. Corn, wheat, rice, and other basic crops make up the bulk of our diet. AI was used in this study to anticipate the consumed yields throughout Saudi Arabia. Ten of the most often grown crops were included. Regression was an issue we had to deal with. The datasets contained crop yields, temperature, insecticides, and rainfall. For the crop yields of maize, potatoes, rice, sorghum, and wheat, we collected data between 1994 and 2016. The dataset is available at the following link: https://www.kaggle.com/code/kushagranull/crop-yield-prediction/notebook.

**Figure 2:**
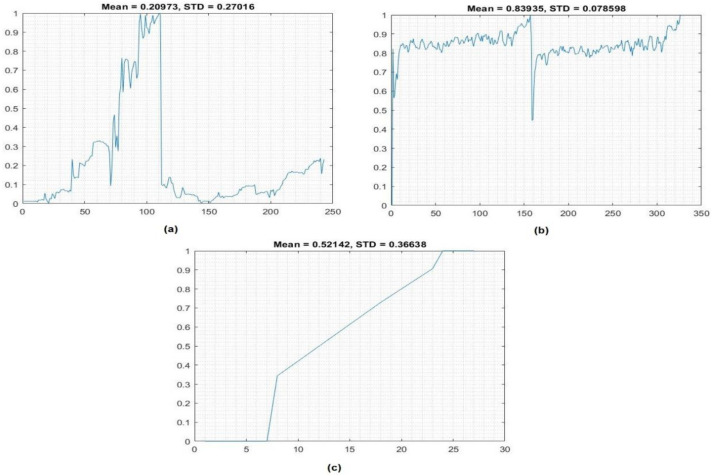
Normalization data: (A) crop yield, (B) temperature, and (C) insecticides.

Figure 2 shows the dataset after normalization, as well as the statistical metrics means and standard division that we calculated. It can be observed that the dataset after min-max normalization values are as follows: mean = 0.20973 and STD = 0.270; the *Y*-axis represents the scaling of data, and the sample identifications are presented on the *X*-axis.

### Normalization method

Normalizing data with min-max normalization is an often-used practice. The minimum and maximum values of each feature are translated into zero and a decimal between 0 and 1, respectively, for each feature.

(1) \begin{eqnarray*}{z}_{n}= \frac{x-{x}_{\mathrm{min}}}{{x}_{\mathrm{max}-{x}_{\mathrm{min}}}} \left( {\mathrm{New}}_{{\mathrm{max}}_{x}}-{\mathrm{New}}_{{\mathrm{min}}_{x}} \right) +{\mathrm{New}}_{{\mathrm{min}}_{x}}.\end{eqnarray*}

The *x*_max and *x*_min are the maximum and minimum values, respectively. New (min_ *x*) is the smallest number, while New (max_x) is the largest number.

### Proposed model

ANNs are a kind of AI that mimics the human brain (Gavahi, Abbaszadeh & Moradkhani, 2021). Densely coupled neurons, the network architecture, and the learning technique influence a neural network’s function. These are simulations of biological brain networks. The ANN technique may help in pattern identification and data classification (Alkahtani & Aldhyani, 2022; Mehedi et al., 2021; Alkahtani & Aldhyani, 2021b; Aldhyani & Alkahtani, 2022). The MLP model is the most often utilized ANN, notably in environmental research. This method may be used to match features and solve pattern recognition problems. MLP may also be used to categorize various linear patterns. These are feedforward neural networks (FNNs) that include several layers of units between the input and output layers. Examples of how a neuron’s output might be expressed are as follows:

(2) \begin{eqnarray*}\xi =\sum _{i=1}^{n}{w}_{i}{x}_{i}-b={w}^{T}x-b\end{eqnarray*}

(3) \begin{eqnarray*}y=\sigma (\xi )\end{eqnarray*}

(4) \begin{eqnarray*}\sigma \left( \xi \right) = \frac{1}{1+{\mathrm{e}}^{-(\xi )}} ,\end{eqnarray*}

where *xi* is the number of the *i*th input, *w*_*i*_ isthe link weight from the ith input, *w* = (*w*_1_…*w*_*n*_)*T* is the total weight, where *x*_*i*_ is the number of the *i*th input (*x*_1_…*x*_*n*_). A threshold or bias is denoted by the letter *b*, while the number n indicates the total number of inputs. The job of the activation function *s*(*x*) is typically to transfer the real numbers into the interval, and this duty can be performed by a continuous or discontinuous function. It is also possible to utilize the sigmoidal activation function. It is possible to express it using the form (Alkahtani & Aldhyani, 2021a).

Training (learning), testing, and validation helped to improve the network design. The sum-of-squares error function was used to evaluate the performance of each neural network in the subsequent stages of model creation. The ANN training method involved an iterative adjustment of the strength of connections between neurons in adjacent layers and the parameters of activation functions. An attempt was made to reduce the training error (El) using the training data set (Chen, 2020). The test error (Et) was also determined for each iteration of the training process to evaluate its accuracy. Network overtraining can occur when E1s cease decreasing or when they decrease but Ets increase, which usually implies overtraining. This can be detected by looking for an increase in E1s and a decrease in Ets. The Levenberg–Marquardt function was used to train the neural networks.

### Model performance

Different MLP neural network models and their practical appropriateness were evaluated using statistical criteria for prognostic model validation in this study. The models’ accuracy in terms of fitting was assessed using the coefficient of determination (R2). In this study, the normalized root mean square error (NRMSE), mean square error (MSE), and root mean square error (RMSE) were used to calculate the average absolute difference between forecasts and observations.

(5) \begin{eqnarray*}\mathrm{MSE}& = \frac{1}{n} \sum _{i=1}^{n}({y}_{i,\mathrm{exp}}-{y}_{i,\mathrm{pred}})^{2}\end{eqnarray*}

(6) \begin{eqnarray*}\mathrm{RMSE}& =\sqrt{\sum _{i=1}^{n} \frac{{ \left( {y}_{i,\mathrm{exp}}-{y}_{i,\mathrm{pred}} \right) }^{2}}{n} }\end{eqnarray*}

(7) \begin{eqnarray*}R\text{%}& = \frac{n \left( \sum _{i=1}^{n}{y}_{i,\mathrm{exp}}\times {y}_{i,\mathrm{pred}} \right) - \left( \sum _{i=1}^{n}{y}_{i,\mathrm{exp}} \right) \left( \sum _{i=1}^{n}{y}_{i,\mathrm{pred}} \right) }{\sqrt{ \left[ n{ \left( \sum _{i=1}^{n}{y}_{i,\mathrm{exp}} \right) }^{2}-{ \left( \sum _{i=1}^{n}{y}_{i,\mathrm{exp}} \right) }^{2} \right] \left[ n{ \left( \sum _{i=1}^{n}{y}_{i,\mathrm{pred}} \right) }^{2}-{ \left( \sum _{i=1}^{n}{y}_{i,\mathrm{pred}} \right) }^{2} \right] }} \times 100\end{eqnarray*}

(8) \begin{eqnarray*}{R}^{2}& =1- \frac{\sum _{i=1}^{n}({y}_{i,\mathrm{exp}}-{y}_{i,\mathrm{pred}})^{2}}{\sum _{i=1}^{n}({y}_{i,\mathrm{exp}}-{y}_{\mathrm{avg},\mathrm{exp}})^{2}} \end{eqnarray*}

(9) \begin{eqnarray*}\mathrm{NRMSE}= \frac{\sqrt{ \frac{1}{n} \sum _{k=1}^{n}({y}_{i,\mathrm{exp}}-{y}_{i,\mathrm{pred}})^{2}}}{{y}_{i,\mathrm{pred}}} .\end{eqnarray*}

In this case, the *y*_*i*,exp_ represents the experimental value of the data point *i* and *y*_*i*,pred_ is the predicted value of the data point *i*. The *y*_avg,exp_ is the represented average of the experimental values, and *n* is the total training values

## Experiments

Models involving densely interconnected structures in which numerous interactions exist and where there is no basis for linear approximation are some of the most powerful approaches to solving engineering problems. Complex system modeling could benefit from the use of ANNs, which have been shown in numerous studies to have excellent prediction accuracy, generalization capacity, and robustness to noisy input. In this study, we attempted to use MLP to construct a prediction model for modeling and predicting the crop yields in Saudi Arabia.

As previously noted, an efficient MLP model was created based on the datasets gathered from different regions in Saud Arabia. The computational platform used for the modeling was MATLAB 2020. The input variables employed for the modeling were crop yield, temperature, rainfall, and insecticides. The Min-Max approach was employed for standardizing the data. The prediction performance of the constructed model was assessed using three statistical metrics: MSE, RMSE, and R2.

### Development of the MLP model

The MLP model contains an input layer, a hidden layer, and an output layer; the first two have 15 neurons, while the output layer contains one neuron. The input variables are represented by the number of neurons in the input layer, while the projected output value is represented by a single neuron in the output layer. It is utilized for cross-validation of the prediction model, which is further constituted of two hidden units and executes computational tasks. In training, one unit is employed; in validation, trainlm is a network training function that uses Levenberg–Marquardt optimization to update weight and bias values in the network. With a loss function that is the sum of squared errors, the Levenberg–Marquardt algorithm is best suited for this use.

**Figure 3:**
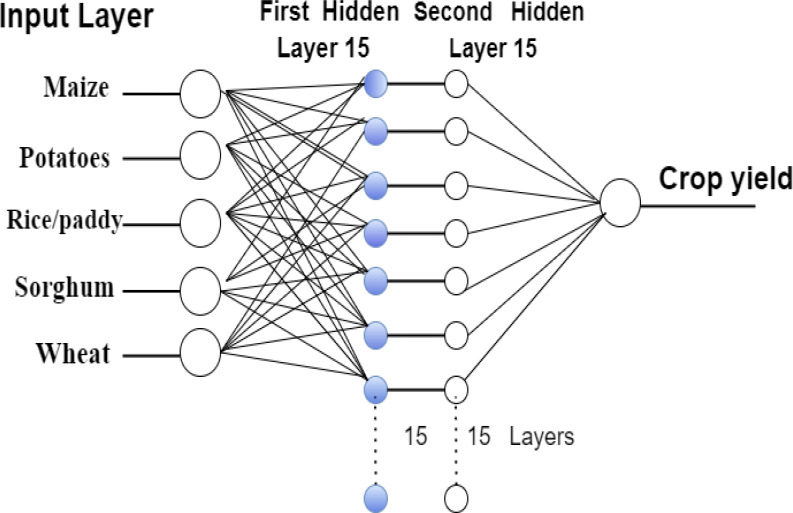
The topology of the MPL model for crop yield.

Figure 3 depicts the MPL model for predicting crop yields in Saudi Arabia, while Table 1 lists the model’s parameter values.

**Table 1:** Parameters of the developed MLP model.

First hidden layer	15
Second hidden layer	15
Input layer	4
maxIterations	100
Maximum number of epochs	70
Delays	[1 2 5 7]
Gradient	1.26
Validation check	6

### Training process of the MLP model

**Figure 4:**
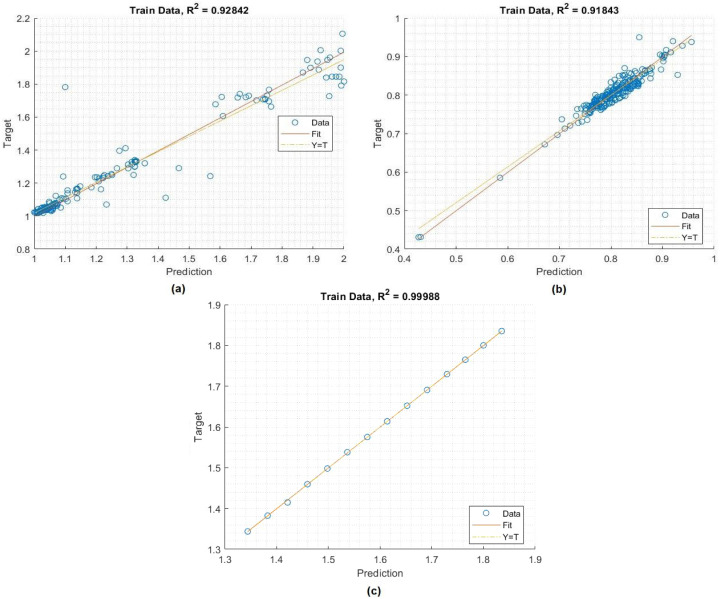
The topology of the MPL model for crop yield. Performance of the MLP model in the training process: a) crop yield, b) temperature, c) insecticides.

Training is a key step in the development of a highly effective model based on some experimental data. Of the total datasets, 70% were used at this stage for this purpose. As can be seen in Fig. 4 and Table 2, the constructed MLP model performs admirably in terms of the evaluation metrics. The correlation between the prediction values and the crop yield parameters values is presented. The MLP model has high values of *R* (>100%) for predicting insecticide values and *R*2 (>0.9284) for predicting the crop yield, in addition to low values of MSE, RMSE, and NRMSE. These values show that the system has been optimized to meet the specified objectives.

**Table 2:** Performance of the MLP model in the training stage.

Dataset	MSE	RMSE	NRMSE	*R*	*R* ^2^
Crop yield	0.00381	0.06173	0.04493	96.2	92.84
Temperature	0.00270	0.01643	0.02038	93.26	91.84
Insecticides	2.8236 × 10^−06^	0.00168	0.00105	100	91.18

**Figure 5:**
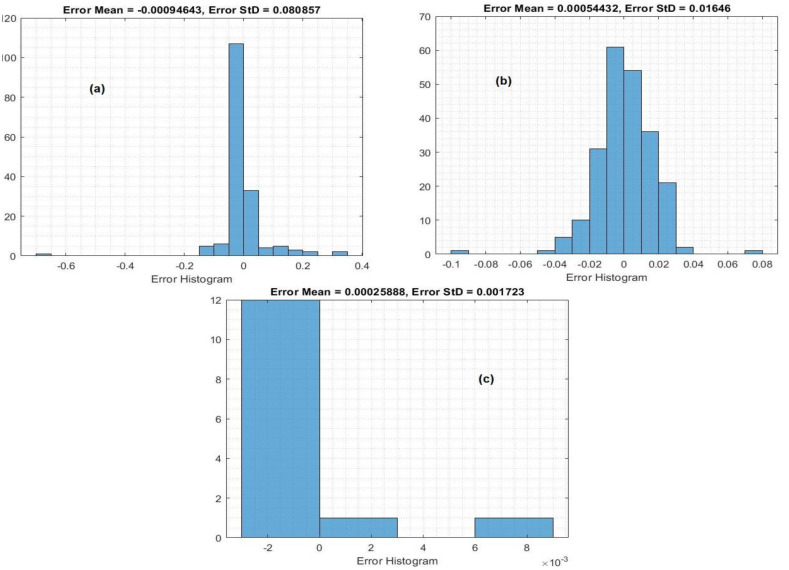
Histogram of the MLP model in the training process: (A) crop yield, (B) temperature, (C) and insecticides.

The histogram inaccuracy of the predicted values at the training state is depicted in Fig. 5. To determine the amount of deviation that exists between the predicted values and the target values, the error histogram metrics were examined. Because these error values explain how the anticipated values differ from the target values, these values can be negative. Also, it specifies how the predicted values deviate from the target values. It was reported that the greatest errors were 0.000946, 0.000544, and 0.000258 for the crop yield, temperature, and insecticides, respectively.

### Testing process of the MLP model

**Figure 6:**
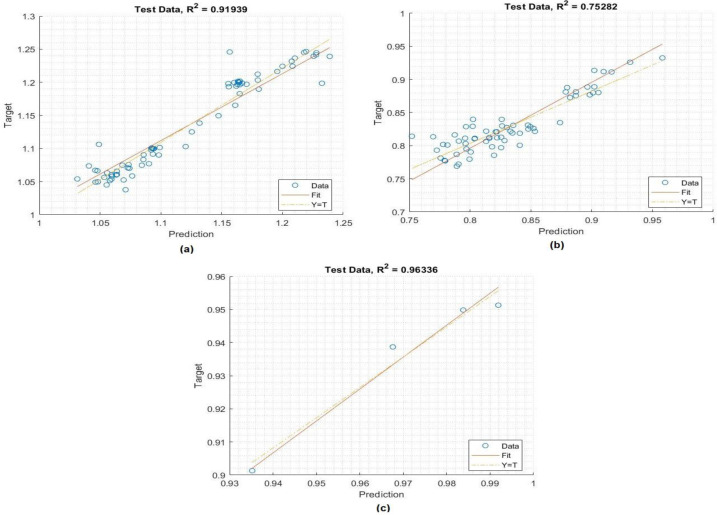
Histogram of the MLP model in the training process: (A) crop yield, (B) temperature, (C) and insecticides.

To verify the accuracy of the MLP model, the testing phase utilized 30% of the datasets, which consisted of previously undisclosed data. The results of the MLP model performance in the testing stage are shown in Table 3, respectively. As can be seen in Fig. 6, there is an outstanding agreement between the values that were predicted and the values that were sought to be found through experimentation. In addition, it was found that the values of R% were very high (100%), and the value of *R*^2^ was very high (96.33%), for predicting insecticides while the values of MSE and RMSE were very low (0.0045 and 0.021 respectively) for predicting temperature values. These demonstrate that the MLP model that was created to forecast the crop yield, temperature and insecticides and it is solutions is both accurate and reliable.

**Table 3:** Results of MLP compare with different prediction results of food security system.

Dataset	MSE	RMSE	NRMSE	*R*	*R* ^2^
Crop yield	0.000548	0.02341	0.02087	92.16	91.93
Temperature	0.004512	0.02124	0.0259	81.05	75.28
Insecticides	0.00111	0.03340	0.0170	100	96.33

**Figure 7:**
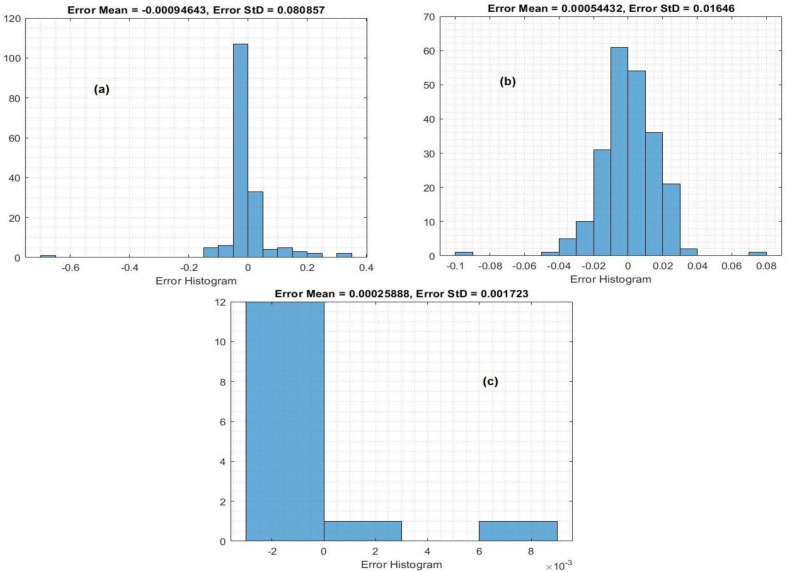
Histogram of the MLP model in the testing process: (A) crop yield, (B) temperature, and (C) insecticides.

Figure 7 shows the histogram inaccuracy of the MLP model during testing for forecasting crop yield. Histogram errors are metrics that are employed to determine the discrepancies between the observed and predicted data. The mean errors in the histograms were 0.000094, 0.00544, and 0.00025 for the crop yield, temperature, and insecticides, respectively.

**Table 4:** Results of MLP compare with different prediction results of food security system.

Ref	Model	Crop yield	Region	Results
Khaki & Wang (2019)	Deep neural network	Maize	United States and Canada	RMSE = 12.18 *R* = 84.01
Pham et al. (2022)	Principal component analysis (PCA) and M	Rice-	Vietnam	RMSE = 5%–12%
Gong et al. (2021)	Temporal convolutional network (TCN) and recurrent neural network (RNN)	Tomato	Newcastle, UK	RMSE = 10.45
Khaki, Wang & Archontoulis (2020b)	(CNN-RNN)	Corn and soybean	United States	RMSE = 8%–9%
Our model	Multilayer perceptron (Proposed system)	Maize, potatoes, rice, sorghum, and wheat	Saudi Arabia	RMSE = 0.04493 *R* = 96.02

### Analysis of the important parameters for increasing the crop yield in Saud Arabia

Our economy and sustainable growth depend on accurate forecasts of agricultural production, which is why crop production forecasting has become a major concern. It aids farmers and the government in developing better post-harvest management in terms of transportation, storage, and distribution at the local, regional, and national levels. Agricultural production optimization and intensification face numerous challenges, one of which is predicting crop yields. Natural conditions can have a considerable impact on crop selection, crop rotations, applied agrotechnical approaches, and long-term land use planning. All these items are essential components of systems that assist farmers in making knowledgeable, expert decisions.

One of the most important aspects of the AI approach is the requirement for a large enough number of training examples based on high-quality observations of a complicated system. The precision of training in intelligent systems is closely related to the amount of information provided and the dependability of that information.

**Figure 8:**
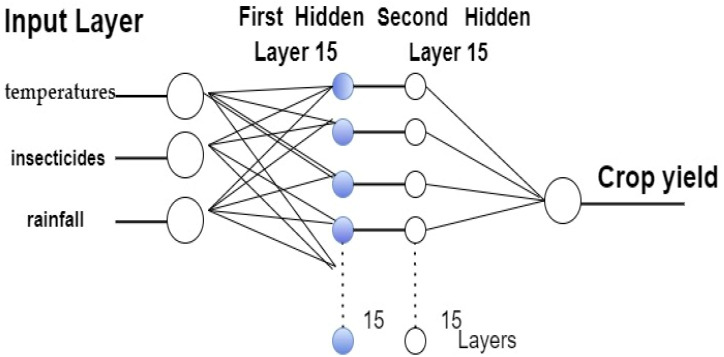
Structure of the MLP for finding the regression between rainfall, temperatures, insecticides, and different crop yields.

To estimate the effect of meteorological conditions of the previous year on the yield of the current year in Saudi Arabia, we employed a vector formed of the average monthly values of rainfall, temperatures, and insecticides. These three essential elements have a greater impact on Saudi Arabia’s crop harvest than others, but many factors affect the agricultural output in Saudi Arabia. Thus, long-term stationary experiments are utilized to determine them. Figure 8 displays the structure of an MLP model for determining the effects of rainfall, temperature, and insecticides on different crop yields, such as those for potatoes, rice, sorghum, and wheat.

**Figure 9:**
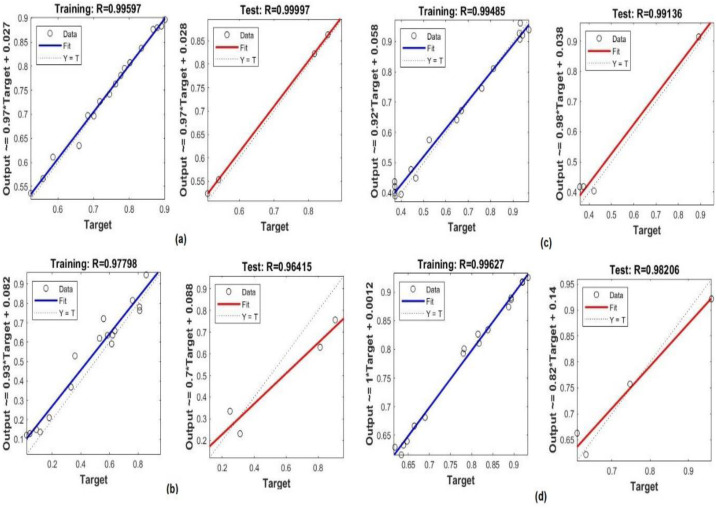
Performance of the MLP model for finding correlations between temperature and crop yields for (A) potatoes, (B) rice, (C) sorghum, and (D) wheat in the training and testing processes.

We also used the MLP model to determine the relationship between crop yields and these parameters to formulate appropriate recommendations for agricultural technology for the upcoming year, taking into account the collected experience and weather conditions monitored over time. Hence, we have looked into the possibility of predicting crop yields using AI. We have applied the MLP model to determine the relationship between the temperature, insecticides, and rainfall amounts with different crop yields, such as potatoes, rice, sorghum, and wheat. Figure 9 shows the regression plot of the MLP model for finding the correlation between temperatures and crop yields It can be observed that temperature had more influence on the crop yields in Saudi Arabia. The MLP score was *R* > 98% for all the crops.

**Figure 10:**
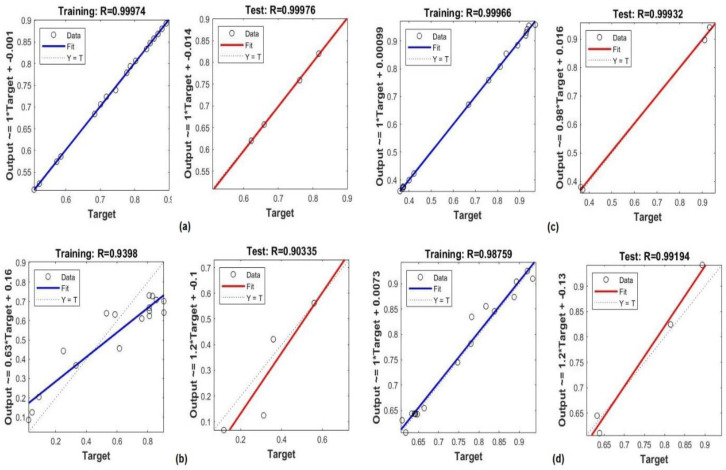
Performance of the MLP model for finding correlations between insecticides and the crop yields for (A) potatoes, (B) rice, (C) sorghum, and (D) wheat in the training and testing processes.

Figure 10 shows the regression between insecticides and crop yields for potatoes, rice, sorghum, and wheat. It shows that insecticides had the most influence on increasing crop yields in Saudi Arabia. The MLP model had the highest regression scores (between *R* > 90% and 99%) for crop yields in the testing and training processes.

**Figure 11:**
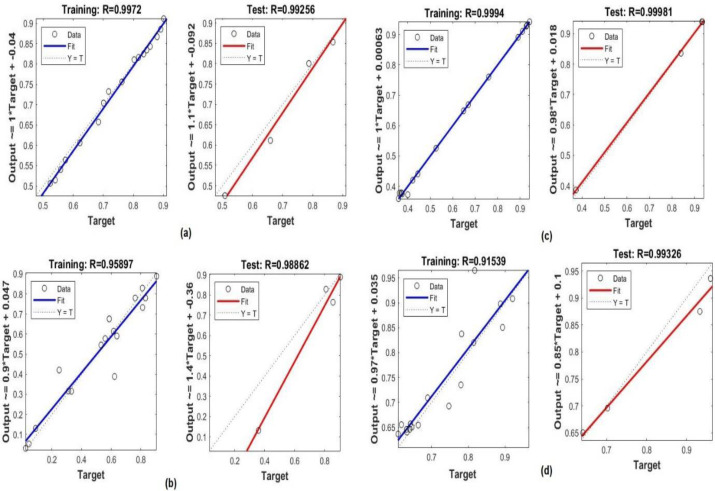
Performance of the MLP model for finding correlations between insecticides and crop yields for (A) potatoes, (B) rice, (C) sorghum, and (D) wheat during the training and testing processes.

Temperatures in Saudi Arabia (SA) can vary widely from place to place and depending on the time of year. Spring and winter have the largest incidences of rainfall, according to an analysis. The rainfall is an important resource for increasing the percentage of the predicted crop yield. Therefore, we have applied the MLP model to determine the relationship between rainfall and crop yields for rice, sorghum, and wheat. Figure 11 shows the regression graph, which shows that rainfall helps increase the crop yield. The scores were *R* > 91% for the testing phase and *R* > 98% for the training phase.

According to the findings of this research project, the effect of weather conditions from the previous year, such as rainfall, temperature, and the use of insecticides, on the yield of the current year is comparable to the overall effect of the agricultural practices that are implemented in Saudi Arabia. Therefore, to accurately predict crop yields for the subsequent agricultural period, it is essential to take into account not only the agricultural practices that are going to be utilized but also the anticipated temperature range, amount of precipitation, and composition of insecticides. Table 4 shows the MLP result against different existing food security systems.

## Conclusion

In agriculture, predicting crop yields is critical. An accurate record of crop yields is vital for making risk management decisions in agriculture. Crop productivity has been the subject of numerous studies utilizing a variety of data mining approaches. Crop yield prediction accuracy, however, has not improved, and AI models have been developed to overcome the difficulties. We created an MLP-based framework to anticipate crop yield in Saudi Arabia utilizing temperature, pesticides, and rainfall to examine the performance of AI models for ecological challenges, especially when temporal and spatial correlations are found in the data. The following conclusions can be derived from this study’s positive findings:

•  An efficient MLP model was successfully developed to predict crop yields, temperature, and insecticides, and high values of *R*% and *R*^2^ with low values of MSE/RMSE were reported for the training and testing phases.

•  The MLP model investigated the relationship between the crop yield types, including potatoes, rice, sorghum, and wheat, with environment parameters, namely, temperature, pesticides, and rainfall. Temperature, pesticides, and rainfall were effective in increasing the product crop yield in Saudi Arabia.

•  This research shows that an AI model may be used to estimate agricultural yields based on a variety of environmental factors. New agricultural techniques that help to attain more sustainable and secure food production could be developed based on the results in this study.

•  More complex models that are more accurate and easier to understand will be a focus of our future research.

##  Supplemental Information

10.7717/peerj-cs.1104/supp-1 Supplemental Information 1 Code

## Additional Information and Declarations

### Data Availability

The following information was supplied regarding data availability: The code is available in the Supplemental File. The data is available at Kaggle: https://www.kaggle.com/code/kushagranull/crop-yield-prediction/notebook.
